# Cold stress triggers premature fruit abscission through ABA-dependent signal transduction in early developing apple

**DOI:** 10.1371/journal.pone.0249975

**Published:** 2021-04-09

**Authors:** Youngsuk Lee, Van Giap Do, Seonae Kim, Hunjoong Kweon, Tony K. McGhie

**Affiliations:** 1 Apple Research Institute, National Institute of Horticultural and Herbal Science, Rural Development Administration, Gunwi, South Korea; 2 School of Biological Sciences, College of National Science, Seoul National University, Seoul, South Korea; 3 The New Zealand Institute for Plant and Food Research Limited, Palmerston North, New Zealand; Iwate University, JAPAN

## Abstract

Fruit abscission is a complex physiological process that is regulated by internal and environmental factors. During early development, apple fruit are exposed to extreme temperature fluctuations that are associated with premature fruit drop; however, their effect on fruit abscission is largely unknown. We hypothesized that fruit abscission is triggered by cold stress and investigated the molecular basis of premature fruit drop using RNA-Seq and metabolomics data from apple fruit undergoing abscission following cold stress in the field. Genes responsive to abscisic acid signaling and cell wall degradation were upregulated during abscission, consistent with the increased abscisic acid concentrations detected by liquid chromatography-mass spectrometry. We performed *ex vivo* cold shock experiments with excised tree subunits consisting of a branch, pedicel, and fruit. Abscission induction occurred in the cold-stressed subunits with concurrent upregulation of abscisic acid biosynthesis (*MdNCED1*) and metabolism (*MdCYP707A*) genes, and ethylene biosynthesis (*MdACS1*) and receptor (*MdETR2*) genes in the pedicel. Another key finding was the activation of cytoplasmic streaming in abscission-zone cells detected by electron microscopy. Our results provide a novel insight into the molecular basis of fruit abscission physiology in response to cold stress in apple.

## Introduction

Fruit abscission is a complex process that is regulated by internal and external factors. During the abscission process, the abscission-zone (AZ) tissues are induced, which leads to cell separation. The abscission signals are regulated by hormones, such as ethylene and abscisic acid (ABA), and signal transduction pathways [1]. The formation of AZ cell layers is induced by the *JOINTLESS* and *MACROCALYX* genes [2], accompanied by the modification of cell wall components such as lignin and pectin [3]. Previous studies have mainly focused on elucidating the intrinsic factors of the abscission signal including fruitlet abscission or pre-harvest drop [4–6]. However, the abscission studies on fruit at early developing stage affected by environmental stresses have rarely been reported.

The cold response of trees is mediated by C-repeat binding factor (CBF) signaling [7–9], reviewed in [10]. During the cold stress response, the hormone ABA is synthesized, which can activate the CBF-mediated signals [11]. Subsequently, the expression of cold-responsive (*COR*) genes is amplified, and as a result, trees undergo various physiological changes involving cell dehydration correlated with dehydrins [12].

Apple (*Malus* × *domestica* Borkh.) is one of the major perennial fruit crops that occupies a large portion of the world fruit industry. Extreme changes in weather conditions are expected to increase in frequency due to climate change [13]. Orchard trees are exposed to abnormally low temperatures in the primary apple production regions in Korea, and the frequency of this trend is expected to further increase according to the prediction model using regional climate change scenarios [14]. During early stages of development, fruit can be exposed to extreme changes/fluctuations in temperature in spring; however, the mechanism underlying premature fruit drop affected by the environmental stress is largely unknown. Changes in temperature conditions of the orchard may cause fruit drop if the abscission signal transduction is induced. Yuan and Burns [15] demonstrated that exposure to varying temperatures contributes to different responses of leaf and fruit abscission under ethylene application. Unlike the natural abscission of the excessive fruitlet clusters of trees bearing too many fruit, the unexpected fruit drop during the normal fruit development can decrease the yield of the orchard. Therefore, from the perspective of the fruit industry, it is important to understand the abnormal fruit abscission mechanism triggered by environmental factors such as cold stress.

**Fig 1:**
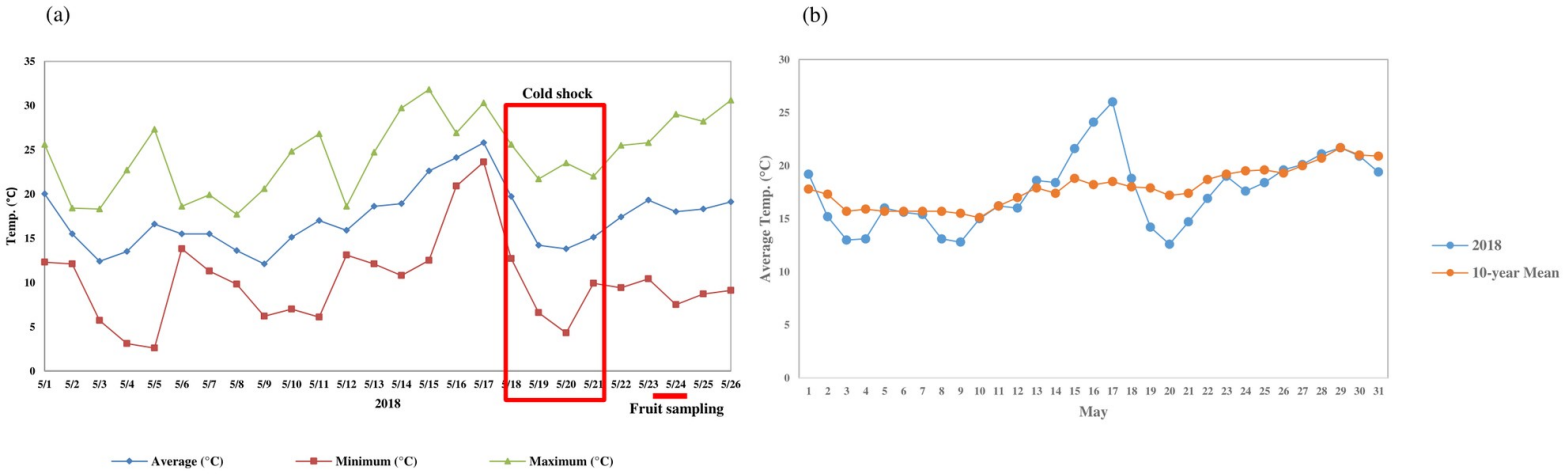
Meteorological data at the experimental plot of the Apple Research Institute, Gunwi (lat. 36.28°N, long. 128.47°E). (a) Change in daily minimum, mean, and maximum temperature during May of 2018. (b) Comparison of daily mean temperatures to 10-year annual mean.

In late May 2018, abnormal fruit drop was reported in many orchards in the apple production areas of South Korea. This damage was also observed at the Apple Research Institute (Gunwi; 36.28°N, 128.47°E) following 2 days of cold stress and a sharp fluctuation in daily mean temperature compared with the 10-year annual mean (Fig 1). In this study, we hypothesized that cold stress induced the abscission of early developing fruit after cold exposure in the field. To test this hypothesis and elucidate the molecular basis of premature fruit drop, we conducted RNA-Seq and metabolomics analyses from young apple fruit collected in the field and investigated the morphological traits of AZ cortical cells by electron microscopy and the tissue-specific quantitative reverse transcription PCR (qRT-PCR) of excised tree subunits consisting of a branch, pedicel, and fruit in *ex vivo* cold shock experiments.

## Materials and methods

### Plant material and sample collection

Six-year-old ‘Hongro’ apple scions grafted onto ‘M9’ rootstocks (Hongro/M9) were grown under field conditions at the Apple Research Institute, Gunwi, South Korea. Trees were managed according to the standard field management of a conventional slender spindle apple orchard with the stable fruit load management of flower/fruitlet thinning followed by the winter pruning practices. To investigate the changes in fruit during the abscission process in the field before any additional physiological changes after fruit fall, we collected 3 cm diameter young fruit with the pedicel (hereafter, referred to as fruit/pedicel) samples on 24th May (37 days after full bloom, DAFB) for RNA-Seq and metabolomics analyses to compare the physiological responses under abscission (S1 Fig). The following year, Hongro/M9 apple subunits consisting of a branch, pedicel, and fruit were excised for *ex vivo* cold shock experiment and placed into a floral foam to prevent dehydration. These subunits were incubated at 25 °C following the initial 2 hr cold shock treatment and sampled after the incubation at two early time points (6, 18 hr) for RNA extraction and one late time point (168 hr) for microscopic analysis. To minimize any effects due to individual variations, we collected the samples from a single Hongro/M9 tree for experiments in both years. In May 2020, we collected tree subunits of Hongro/M9 apple trees at 42 DAFB for the second *ex vivo* experiment. Subunits were divided into four groups by treatments as follows: Control (water); ABA (125 mg/L of ABA); Cold (initial cold shock at 4 °C for 2 hr); and Cold + ABA (125 mg/L ABA followed by initial cold shock at 4 °C for 2 hr). The exogenous ABA treatment was applied onto the branch tissues closely connected to pedicel. All groups were incubated at 25 °C and sampled after the incubation at four early time points (0, 2, 12, 24 hr) for RNA extraction and one late time point (168 hr) for microscopic analysis.

### Weather data

Meteorological data were downloaded from the Korean Meteorological Administration (http://www.kma.go.kr). Data included daily mean, minimum, and maximum air temperature in May 2018 and the annual mean temperature for 10 years at the Apple Research Institute.

### RNA extraction and bioinformatics analyses

Total RNA was extracted using a modified CTAB method [16]. In the first in-field sampling (2018), we extracted RNA from fruit/pedicel tissues (pooled from four biological replicates) for RNA-Seq and metabolomics analyses. In the next two sampling of *ex vivo* experiments (2019–2020), RNA was extracted from each tissue of tree subunits (pooled from eight biological replicates) for qRT-PCR and microscopic analyses. The concentration of RNA was measured using BioDrop μLITE spectrophotometer (Cambridge, UK).

### RNA sequencing and bioinformatics analyses

Samples with RNA integrity number 7.5 or above were used for RNA-Seq library construction. A total of six libraries were constructed and used for bioinformatics analyses. Detailed methods were provided in the S1 Text. All sequencing data were deposited in the National Center for Biotechnology Information Sequence Read Archive database bearing the BioProject ID PRJNA613278.

### Ultra-high-performance liquid chromatography-mass spectroscopy (UPLC-MS)

The metabolites of lyophilized samples of fruit/pedicel were measured using UPLC-quadrupole-time-of-flight MS (UPLC-QTOF-MS). There were four biological replicates. Metabolites were detected and labelled by accurate mass *m*/*z* and liquid chromatography retention time. Detailed methods are described in S1 Text.

### Quantitative reverse transcription PCR (qRT-PCR)

First-strand complementary DNA was synthesized using 1.0 μg of total RNA, oligo dT primer, and Transcriptor Reverse Transcriptase (Roche, Penzberg, Germany). qRT-PCR analysis was performed in LightCycler 480 SYBR Green I Master mix (Roche, Germany) on a Roche 480 LightCycler^®^ (Basel, Switzerland). Complementary DNA (1:20 dilution) was used as a template (5 μL) in a 20 μL reaction volume. For each sample type, there were four to six technical replicates. PCR cycles were as follows: initial denaturation at 95 °C for 5 min, followed by 45 cycles of 95 °C for 10 s, 65 °C for 15 s, and 72 °C for 12 s, and a final melt curve analysis to determine the amplification of a single product. Primers were designed using Primer-Blast (http://www.ncbi.nlm.nih.gov/tools/Primer-Blast/) to span an intron if possible, with 100–150 bp product size (S1 Table). *MDP0000336547* was selected as the reference gene [17]. Primer efficiencies and relative expression levels of targets were calculated using the Roche 480 Light Cycler software E-Method [18].

### Microscopic analyses

Pedicel tissues of *ex vivo* experiments were sectioned vertically, and photographs were taken using an Olympus SZX16 microscope under bright field. We observed AZ-containing cortical cells using a Carl Zeiss LIBRA 120 transmission electron microscope (Oberkochen, Germany) operating at an acceleration voltage of 120 kV. Detailed methods for transmission electron microscopy analysis are provided in S1 Text.

## Results

### Outcome of cold stress followed by a large temperature fluctuation at in-field site

In late May 2018, unusually high number of fruit drops occurred after a short period of cold in primary apple production regions of South Korea. In the experimental plots of the Apple Research Institute, Gunwi, we observed similar damage with a distinctive yellow peel compared to normal fruit (S1 Fig). After the daily minimum temperature reached a peak of over 20 °C on the 17th May, it decreased rapidly to 4 °C until 20th May (Fig 1a). The comparison of the daily mean temperature data against the 10-year annual mean revealed large temperature fluctuations, from a maximum of +7° on the 17th and -4° on the 20th May (Fig 1b). This indicated an unusual cold shock starting from 17th to 20th May, which might have induced an abnormal fruit drop of early developing apple fruit afterward.

### Differentially expressed genes (DEGs) functionally involved in cell wall modification, drought response, and ABA and ethylene hormone signals in fruit undergoing abscission following cold stress

Using the list of DEGs from the RNA-Seq results, we established the profile of 92 selected DEGs and nine functional groups (S1c Fig). In the cell wall modification class, genes encoding pectinesterase (*MD08G1195600*, *MD04G1008100*, etc.), polygalacturonase (*MD07G1011600*, *MD10G1179100*), and expansin (*MD06G1195100*) were differentially expressed in the fruit under abscission.

DEGs related to oxidation-reduction included some downregulated polyphenol oxidases (e.g., MD10G1298700) and a few upregulated ribulose biphosphate carboxylases (RuBPC) (e.g., *MD13G1041900*). This category also included genes encoding UDP-glucuronosyl/UDP-glucosyltransferase homologs.

The senescence-related DEGs included the downregulated *senescence regulator 40* (*S40*; *MD10G1269600*, *MD00G1093200*) and the upregulated desiccation protectant protein homolog (*MD01G1072000*). The upregulation of both *dehydrin 1* (*DHN1*; *MD02G1140100*) and *LEA14* (*MD 00G1127700*) homologs indicated drought stress response of fruit under abscission; *dehydrin xero 1* (*XERO1*) was downregulated.

In the DNA-binding group, most DEGs were members of transcription factor families that contained either NAC (NAM, ATAF1/2, and CUC2) or bHLH (basic helix-loop-helix) domain. Interestingly, a *SHORT-ROOT* (*SHR*) homolog (*MDP04G1046000*) was found upregulated in the tissues under abscission.

In the class of phytohormone signal transduction, genes involved in both ABA and ethylene signal pathways were detected. A few ABA signal-related genes were annotated to either ABA metabolism (*MD15G1082600*; *ABA 8’-hydroxylase 4*) or downstream response (*MD10G1029100*). The ethylene receptor *Reversion-to-Ethylene Sensitivity* (*RTE*) *1* homolog (*MD15G1250900*) was upregulated in the ‘Abscission’ group.

### High concentration of ABA hormone and lignin precursors in early developing apples undergoing abscission after cold stress

Next, we performed UPLC-QTOF-MS analysis with the same fruit/pedicel samples used for DEG analysis. Twenty-five metabolites were selected and annotated fulfilling criteria of *p* < 0.05 and |log_2_ fold change| > 1 out of the 443 metabolites detected (Table 1). The ABA hormone was highly concentrated in the ‘Abscission’ group. Additionally, lignin precursors, such as quinic acid and ferulic acid, were accumulated more in fruit/pedicel tissues of the ‘Abscission’ group than in control. Other metabolites that significantly differed in concentration between the ‘Abscission’ and ‘Normal’ groups included either flavonoids or amino acids which were subsequently related to the oxidative stress responses. These metabolomics data were consistent with the results of DEG analysis. Therefore, on the basis of both RNA-Seq and metabolomics results combined with the meteorological data, we hypothesized that cold stress affects the abscission of early developing apple fruit.

**Table 1:** Metabolomic profile of young apple fruit/pedicel mixed tissues undergoing abscission.

Metabolite	Adduct	Formula	Annotation	ID confidence^a^	Classification	Comment	p-value	|log2 fold change| ‘Abscission’/‘Normal’	ion detection mode
17.04 min: 487.35 m/z	[2M-H]-	C12H24N2O3	(Iso)Leucyl-leucine	3	Dipeptide	Protein catabolism	0.000	17.71	neg
**15.49 min: 247.133 m/z**	**[M+H-H2O]+**	**C15H20O4**	**Abscisic acid**	**2**	**Terpene**	**ABA biosynthesis, metabolism abscission, cold stress response**	**0.003**	**14.71**	**pos**
1.26 min: 183.086 m/z	[M+H]+	C6H14O6	Sorbitol	2	Sugar alcohol	Product of sugar (galactose) breakdown	0.001	6.91	pos
3.07 min: 268.104 m/z	[M+H]+	C10H13N5O4	Adenosine	2	Nucleic acid	DNA, RNA metabolism	0.006	5.99	pos
2.81 min: 132.102 m/z	[M+H]+	C6H13NO2	(Iso)Leucine	2	Amino acid	Glucose homeostasis, amino acid metabolism	0.016	5.61	pos
1.45 min: 198.097 m/z	[M+NH4]+	C6H12O6	Hexose	2	Sugar	Starch degradation	0.002	3.46	pos
1.26 min: 133.060 m/z	[M+H]+	C4H8N2O3	Asparagine	3	Amino acid	Interconversion of aspartate and asparagine	0.007	2.87	pos
1.30 min: 118.086 m/z	[M+H]+	C5H11NO2	Valine		Amino acid	Amino acid metabolism	0.007	2.87	pos
14.76 min: 871.267 m/z	[2M-H]-	C21H24O10	Phloridzin	2	Polyphenol	Oxidative stress	0.006	2.51	neg
1.27 min: 148.060 m/z	[M+H]+	C5H9NO4	Glutamic acid	2	Amino acid	NAD biosynthesis, oxidative stress	0.005	2.41	pos
10.48 min: 1153.261 m/z	[M-H]-	C60H50O24	Procyanidin tetramer	3	Polyphenol	Oxidative stress	0.000	2.39	neg
1.64 min: 180.086 m/z	[M+H]+	C6H13NO5	Glucosamine	3	Sugar	-	0.000	2.04	pos
**1.44 min: 193.070 m/z**	**[M+H]+**	**C7H12O6**	**Quinic acid**	**2**	**Organic acid**	**Lignin biosynthesis, cell wall component**	**0.014**	**1.60**	**pos**
8.24 min: 305.065 m/z	[M+H]+	C15H12O7	Dihydroquercetin	2	Flavonoid	Oxidative stress	0.000	1.42	pos
**9.02 min: 195.065 m/z**	**[M+H]+**	**C10H10O4**	**Ferulic acid**	**2**	**Phenol**	**Lignin component**	**0.022**	**1.28**	**pos**
16.03 min: 230.248 m/z	[M+NH4]+	C14H28O	Tetradecanal	3	Aldehyde	-	0.008	1.24	pos
10.83 min: 867.213 m/z	[M+H]+	C45H38O18	Procyanidin trimer	2	Polyphenol	Oxidative stress	0.007	1.23	pos
17.04 min: 487.342 m/z	[M-H]-	C30H48O5	Trihydroxyurs-12-en-28-oic acid	2	Terpene	Triterpene	0.001	-1.01	neg
11.46 min: 1729.388 m/z	[M-H]-	C90H74O36	Procyanidin hexamer	3	Polyphenol	Oxidative stress	0.014	-1.08	neg
14.34 min: 447.093 m/z	[M-H]-	C21H20O11	Quercetin 3-rhamnoside	2	Flavonoid	Oxidative stress	0.040	-1.09	neg
1.39 min: 133.014 m/z	[M-H]-	C4H6O5	Malic acid	2	Organic acid	-	0.018	-2.24	neg
14.02 min: 433.077 m/z	[M-H]-	C20H18O11	Quercetin 3-xyloside	2	Flavonoid	Oxidative stress	0.014	-2.28	neg
10.09 min: 865.198 m/z	[M-H]-	C45H38O18	Procyanidin trimer	2	Polyphenol	Oxidative stress	0.013	-3.80	neg
14.94 min: 417.082 m/z	[M-H]-	C20H18O10	Kaempferol 3-arabinoside	2	Flavonoid	Oxidative stress	0.038	-4.72	neg
8.57 min: 577.134 m/z	[M-H]-	C30H26O12	Procyanidin B2	2	Polyphenol	Oxidative stress	0.017	-6.14	neg

Metabolites were detected from UPLC-QTOF-MS analysis with a criteria of *p* < 0.05 and |log_2_ fold change| > 1.

^a^ Metabolites are annotated with three confidence levels (1 = compared to standard; 2 = exact mass/formula + ms/ms or retention time; 3 = exact mass/formula).

### Cytoplasmic streaming of AZ cortical cells evidence for cold-triggered AZ formation

**Fig 2:**
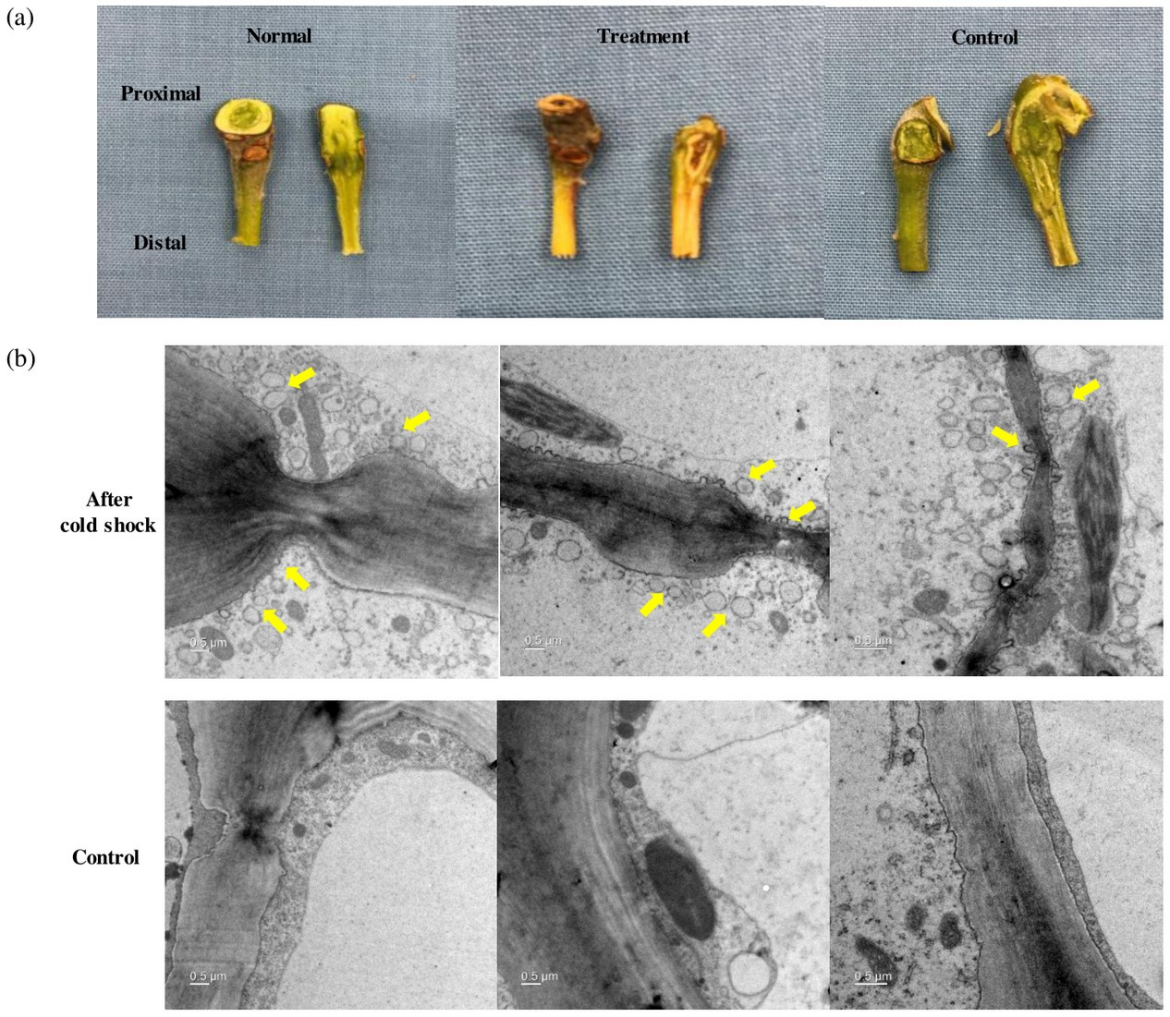
Morphological changes of an excised Hongro/M9 apple tree subunit after *ex vivo* cold shock followed by 168 hr of incubation at 25 °C. (a) Comparison of pedicel tissues containing the abscission zone (AZ) (left: normal, before the incubation; middle: treatment, cold shock followed by the incubation; right: control, after the incubation). (b) Transmission electron microscopy images of the AZ cortical cells at proximal tissues in the pedicel (top: cold treatment, 168 hr of incubation following initial 2 hr of cold shock; bottom: control, 168 hr after incubation without cold shock). Samples were observed with a LIBRA 120 transmission electron microscope at an acceleration voltage of 120 kV, magnification of 4 k (left, middle) and 6.3 k (right). Arrows indicate the development of cytoplasmic vesicles.

In the next year, we conducted an *ex vivo* experiment to investigate whether cold stress contributes to the activation of early fruit abscission signaling. To determine the origin of early responsive signals, we excised tree subunits consisting of a young branch, pedicel, and fruit from Hongro/M9 apple trees (S3 Fig). After one week of incubation followed by 2 hr of cold shock at 4 °C, the AZ-containing pedicels were severely damaged compared with those of the control (Fig 2a). The transmission electron microscopy of the AZ cortical cells revealed that several vesicles were associated with the cytoplasmic streaming in cold-stressed group, but only a few vesicles were detected in the control (Fig 2b). In addition to new vesicles detaching from the cell wall, the thickness of the cell wall was more irregular and variable in the cold-stressed pedicel.

### Tissue-specific response to cold activates ABA signal transduction to potentially induce early fruit abscission

**Fig 3:**
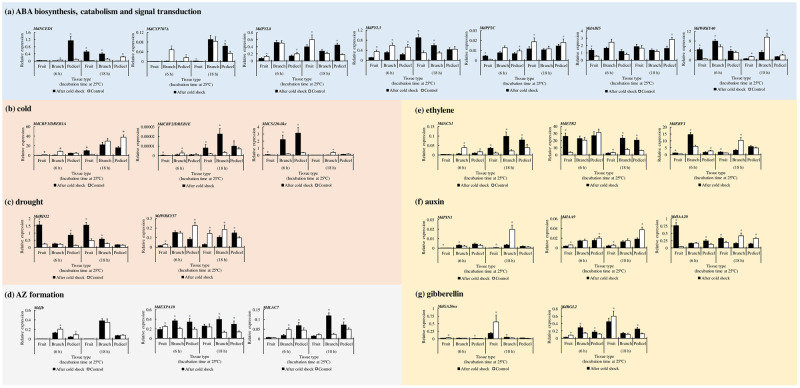
Spatial expression patterns of target genes involved in hormone metabolism and stress response in cold-stressed apple subunit consisting of fruit, branch, and pedicel. (a) abscisic acid (ABA) hormone biosynthesis, catabolism, and signal transduction; (b) Cold stress; (c) Drought stress; (d) Abscission-zone (AZ) formation; (e) Ethylene; (f) Auxin; (g) Gibberellin. *MDP0000336547* was used as reference gene. Data are mean ± standard error (6 ≤ *n* ≤ 8). * indicate significant difference in relative expression between cold treatment and control at *p* < 0.05 by Student’s *t*-test.

To understand the molecular mechanism underlying early cold response and abscission signals, we conducted qRT-PCR on excised tree subunits and compared tissue-specific responses. Within 6 hr of incubation at 25 °C following the initial cold step, the expression of the ABA biosynthesis gene *MdNCED1* in pedicel tissues was upregulated, but then decreased dramatically within the next 12 hr (Fig 3a) whereas a delayed upregulation of the same gene was detected in neighboring fruit and branch tissues. The significant upregulation of *MdCYP707A*, the ABA 8ʹ-hydroxylase, which is a key regulator of ABA metabolism [19], was observed after 18 hr in cold-stressed pedicel.

Next, we investigated the spatial expression pattern of ABA receptor genes in the PYL family (*MdPYL3*, *MdPYL8*). After 18 hr of incubation at 25 °C, the expression of *MdPYL8*, a mediator of the drought stress response [20], increased significantly in the cold-stressed pedicel but not in the fruit or branch. In contrast, *MdPYL3*, which is involved in cold and drought stress response [21], was significantly upregulated in both fruit and branch tissues. In the cold-stressed pedicel, the downregulation of ABA repressor *MdPP2C* was sustained at both 6 and 18 hr of incubation, whereas the branch tissue did not show any significant difference. The ABA Insensitive 5 gene (*MdABI5*) plays a key role in regulating the core ABA metabolism signaling [22, reviewed in 23]. *MdABI5* was significantly upregulated in the fruit within 6 hr. In contrast, its expression was significantly downregulated in the pedicel after 18 hr. Interestingly, the expression of *MdWRKY40*, the repressor of ABA downstream response, was upregulated in all tissue types during the early response (6 hr), but it was significantly downregulated within the next 12 hr.

The CBF family is a key regulator of cold stress response and can also be activated by ABA [11]. In Fig 3b, *MdCBF2* expression, a gene that is known to be highly responsive to cold and to play a key role in CBF regulation [24, 25], increased significantly after 18 hr in the fruit and branch; there was no significant change in the pedicel. As a consequence of cold response, not only is the expression of *COR* genes activated by CBF signals, but the dehydration response is induced as well [reviewed in 26]. We selected CBF downstream targets including one *COR* gene, *Cold shock protein 120* (*CS120*)*-like*, and a couple of drought-responsive genes that differed in expression among tissue types between the cold shock treatment and control (Fig 3c). Within 6 hr after the cold shock, the expression of *MdCS120-like* was upregulated in all tissue types, but more extensively in the branch and pedicel. The expression of the *response to dehydration 22* (*MdRD22*) gene, which is responsive to both ABA and drought stress [27], was significantly upregulated in fruit and the pedicel within 6 hr, as well as in the branch 18 hr later. In contrast, the *MdWRKY57* gene was upregulated after 18 hr in the pedicel and suppressed in both the fruit and branch at the same time point.

### Tissue-specific response to AZ activation through cell wall modification and hormone response signals

Next, we investigated target genes related to cell wall modification, which may be substantially associated with AZ formation (Fig 3d). We selected one gene of each laccase and expansin families as the qRT-PCR target, the two families that were present in the list of DEG analysis in 2018 (S2c Fig). *Laccase 7* (*LAC7*) is involved in lignin polymerization and discerns the non-separating cells from separating cells when plants undergo cell wall processing accompanied by the lignin brace during the abscission [28]. The branch tissue showed the biggest difference in the change in expression from 6 to 18 hr after the initial cold shock. *Expansins* (*EXP*s) are involved in cell wall loosening by disrupting the bond between cellulose and glycans [29]. In our study, *MdEXPA10* expression was significantly upregulated in both the branch and pedicel compared with that in the control. *JOINTLESS* is responsible for AZ formation [2]. Within 6 hr, the expression of *MdJb*, a homolog of *JOINTLESS*, was significantly downregulated in the cold-stressed branch and pedicel, but it did not show a significant change at 18 hr.

Fig 3e–3g illustrates the spatial relative expression of genes involved in hormone signal transduction of ethylene, auxin, and gibberellin. The expression of *ACC-synthase* (*MdACS1*), a gene regulating ethylene biosynthesis, was significantly upregulated in the cold-stressed subunits after 18 hr (Fig 3e). Additionally, both the branch and pedicel tissues revealed a significant difference in the expression of the ethylene receptor (*MdETR2*) at 18 hr. The expression of *ethylene responsive factor 1* (*MdERF1*) was significantly upregulated in fruit and branch tissues at 6 hr.

Auxin-related genes were downregulated as a late response (Fig 3f). The expression of the auxin efflux carrier *MdPIN1* was significantly downregulated in the fruit and branch after 18 hr. Auxin/indole-3-acetic acid (Aux/IAA) family has been shown to repress the transcription of auxin-responsive factors, and to participate in abiotic stress tolerance [30]. We selected *MdIAA9* and *MdIAA20* as target genes because *MdIAA9* is thought to be involved in drought tolerance and *MdIAA20* is related to chilling tolerance in apple [31]. Interestingly, *MdIAA9* expression was maintained significantly downregulated in cold-stressed fruit and pedicel tissues, and *MdIAA20* was significantly downregulated in the branch and pedicel after 18 hr.

We compared the expression of *Gibberellin* (GA) *20-oxidase* (*MdGA20ox*) which regulates GA biosynthesis (Fig 3g). The *MdGA20ox* expression was found to be downregulated in the cold-stressed fruit after 6 and 18 hr. RGL genes which encode the DELLA protein family are thought to be associated with cold stress response in apple [32]. The expression of *MdRGL2* was upregulated in the cold-exposed branch and pedicel at 6 hr, and remained significantly upregulated in the pedicel at 18 hr.

### Exogenous ABA application promotes the development of AZ cells under cold and mediates signals leading to abscission induction

If ABA-dependent signals triggered by cold do contribute to abscission induction, exogenous ABA treatment on excised apple subunits may also lead to the responses of AZ development and expression patterns of relevant genes. To elucidate this, we conducted a second *ex vivo* experiment with ABA and cold stress treatments.

**Fig 4:**
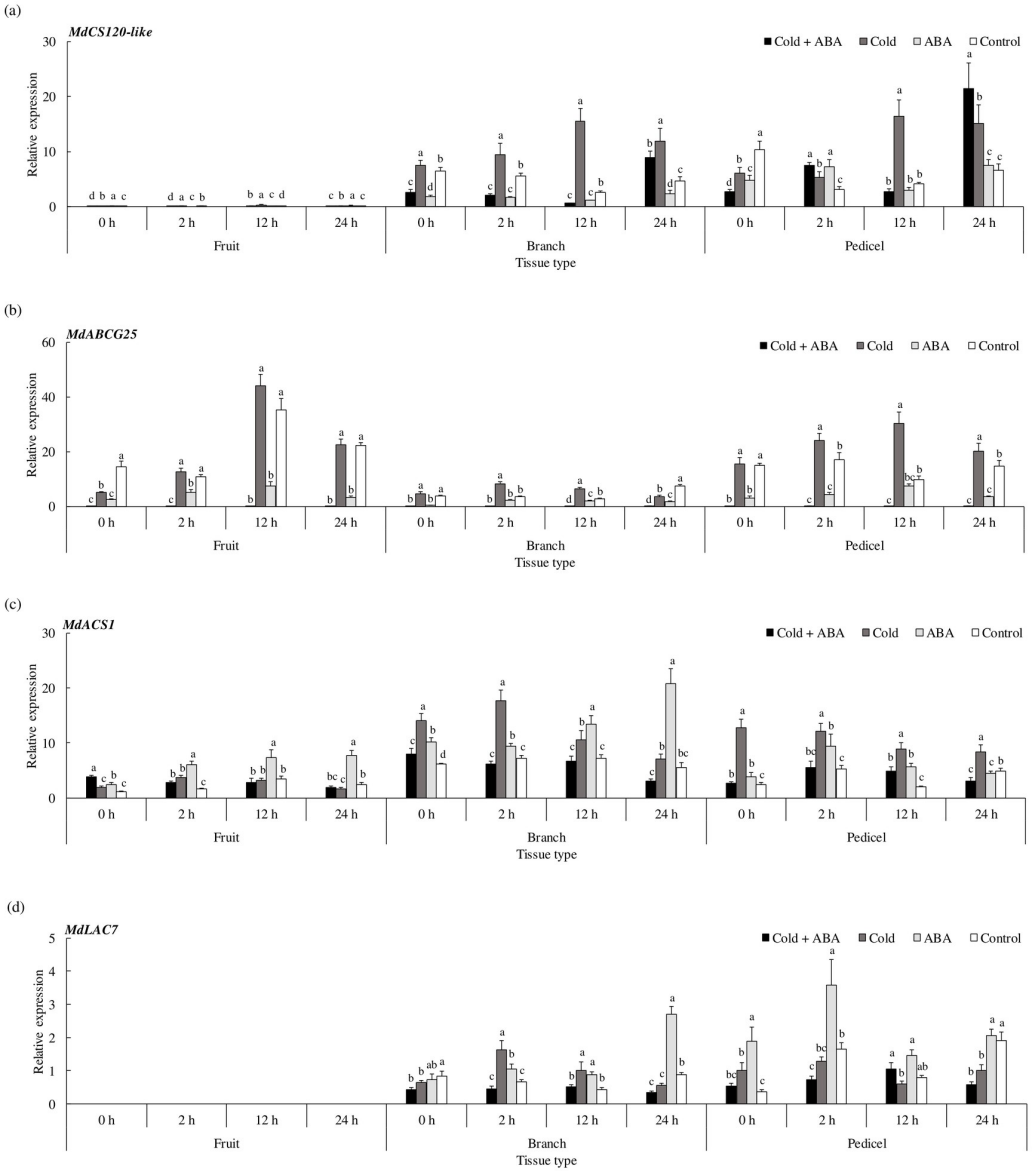
Spatial expression patterns of genes involved in metabolic responses. (a) Cold response, (b) abscisic acid (ABA) transport, (c) ethylene biosynthesis, and (d) cell wall modification in apple subunits consisting of a branch, pedicel, and fruit. Samples were grouped by treatments as follows: Control (water); ABA (125 mg/L of ABA); Cold (initial cold shock at 4 °C for 2 hr); Cold + ABA (125 mg/L ABA followed by initial cold shock at 4 °C for 2 hr). *MDP0000336547* was used as reference gene. Data are mean ± standard error (*n* = 6). Data were analyzed using two-way ANOVA. Values with different letters differ significantly from each other at *p* < 0.05.

First, we measured the expression of *MdCS120-like*, a member of the COR genes responsible for cold response (Fig 4a). The expression of *MdCS120-like* was the highest in pedicel tissues under cold and ABA treatment at 24 hr, while in branch tissues, it was maintained significantly upregulated at all time points in treatments with cold stress without ABA. Next, we selected *ABCG25* as the target gene, an ABA transporter located at the plasma membrane mediating the homeostasis of ABA. Previously, Park et al. [33] demonstrated that the activity of *ABCG25* is controlled by stress conditions. If there were differences in response between cold-induced ABA signaling and that from exogenous ABA application, the expression of the ABA transporter would differ among the groups as well. Indeed, *MdABCG25* expression was significantly upregulated in the cold-stressed pedicel at all time points, whereas ABA application maintained it downregulated regardless of the cold stress (Fig 4b). Then, we compared the expression of *MdACS1* to investigate the gene expression pattern of ethylene biosynthesis. The expression was significantly upregulated in the pedicel under cold at all time points (Fig 4c). Meanwhile, in branch tissue, the expression difference was higher in ABA-treated group compared with that of the cold-stressed group after 12 hr of incubation. In terms of cell wall modification, we investigated *MdLAC7* expression among treatment groups (Fig 4d). Although the significant upregulation appeared in the cold-stressed branch early from 2 hr to 12 hr of incubation, the highest expression was detected after 24 hr in ABA-treated group.

We examined the development of AZ cortical cells at the proximal end of the pedicel in each group (S4 Fig). Interestingly, exogenous ABA application induced the development of cytoplasmic vesicles at similar level to the one in cold stress. The cytoplasmic streaming of vesicles was extensive in both groups under cold and ABA treatments. Taken together, these data indicate that ABA signaling induced by cold may possibly mediate the abscission induction of early developing apple fruit.

## Discussion

### Transcriptomic evidence of cold stress in young apple fruit undergoing abscission

The RNA-Seq analysis provided potential evidence that the apple fruit collected in the latter part of May 2018 not only underwent abscission but was also cold-stressed. For example, the expression of *dehydrin 1* (*DHN1*) homolog *MD02G1140100*, a cold stress marker, was downregulated (S2c Fig). Dehydrins are categorized into the LEA family, which contributes to cold tolerance. Although *MdDHN1* was upregulated in the ‘Abscission’ group, *dehydrin xero 1* (*XERO1*) homolog *MD02G1139900* was rather downregulated. An earlier study on peach demonstrated that *DHN1* responds to low temperature and *XERO1* is induced by drought, not by cold [34]. Therefore, the upregulation of *DHN1* observed in our study is likely the result of the cold stress response. Two *RuBPC* homolog genes in the oxidation-reduction category were upregulated in the ‘Abscission’ group. *RuBPC*s are known to render plants more susceptible to thermal variation in response to chilling stress [35].

### Cold response and the emergence of spatial patterns of ABA signal induction in apple subunits

Unlike mature fruit, apple fruit at early stage of development is mostly seedless or has very few incomplete seeds. Assuming that endogenous ABA is mainly generated from seeds during fruit development [36, 37], ABA-dependent signals for either cold response or abscission induction may originate not merely from fruit, other tissues likely also contribute to the amplification of abscission induction signals.

Indeed, there was a difference in ABA signal induction among tissue types in cold-stressed apple subunits. As a consequence of early cold stress response, the ABA biosynthesis gene (*MdNCED1*) was activated within 6 hr in the pedicel but not in the branch or fruit (Fig 3a). After 18 hr, a delayed upregulation of the ABA biosynthesis gene was observed in branch and fruit tissues, whereas the same gene was downregulated in the pedicel. ABA can be transported to neighboring tissues, where its presence may amplify its biosynthesis [38]. Therefore, the upregulation of *MdNCED1* signals in branch and fruit tissues could be stimulated later by ABA that originated from the pedicel after 6 hr of incubation as a result of the primary cold response.

The results of our study showed that the branch tissues exhibited a relatively high level of cold response compared to other tissues. Initially, both *MdCS120-like* and *MdERF1* were upregulated in the branch. *CS120-like* is a member of COR signaling [39], and *ERF1* is also responsive to cold and is associated with accelerated abscission [40–42]. The CBF signal was detected at 18 hr, which might had been preceded by a signal that activated COR signaling at some point earlier than 6 hr, as suggested by upregulated *MdCS120-like* expression at 6 hr in all tissue types.

Furthermore, we observed that the pedicel not only developed AZ cells during 168 hr of incubation but was also severely damaged by desiccation as a result of *ex vivo* cold treatment (Fig 2). The response of this cold-driven dehydration had occurred at early time points with the increased expression of responsive genes (Fig 3c). In pedicel tissues, *MdRD22*, which is responsive to both ABA and drought stress [27], and *MdWRKY57*, which activates the ABA biosynthesis by binding directly to the NCED promoter [43], were significantly upregulated at 6 hr and 18 hr of incubation, respectively.

### Responses in cell wall modification contribute to AZ formation with the cytoplasmic stream of vesicles

From the 2019 *ex vivo* experiment, we observed that in both branch and pedicel tissues the cell wall loosening (*MdEXPA10*) and the lignin polymerization (*MdLAC7*) signals were upregulated for the substantial AZ formation 18 hr after the initial cold shock (Fig 3d). This is possible because the abscission signal is affected by the cold response. A similar tendency was observed in the RNA-Seq and metabolomics data for *ex vivo* experiment conducted the previous year. Based on the LC-MS results, metabolites related to lignin biosynthesis (quinic acid and ferulic acid) significantly accumulated in fruit/pedicel mixed tissues (Table 1). Transcriptomics data revealed an upregulated *SHR* homolog *MDP04G1046000*, which is related to the lignin signaling pathway for abscission induction (S2c Fig). *SHR* mediates the formation of Casparian strips in endodermis [28]. When the neighboring cells undergo organ separation during abscission, a lignin brace is constructed in the secession cells. Therefore, such *SHR* gene, which mediates the formation of lignin structure, would be positively regulated in fruit pedicel as a consequence of abscission induction following cold stress.

Another member participating in the AZ development is pectin, one of the main cell wall components. A number of pectinesterases were differentially expressed, as shown in S2c Fig. Pectin is accumulated during AZ formation, and its methylesterification was altered before cell separation [44]. Furthermore, AZ cells show the association of cytoplasmic vesicles during the cell separation process (reviewed in [45]). We observed the accumulation of vesicles in the cytoplasm of AZ cortical cells as a result of cold shock (Fig 2b, S4 Fig). Several of them appeared to be bound to the cell wall, and the cytoplasmic streaming of vesicles seemed strongly associated with the modification of the cell wall by carrying molecules to be used in cell wall metabolism or by removing these from the cell wall.

### Spatial responses leading to complex downstream signal transduction for AZ formation

Phytohormone signal transduction affects the abscission process and it is controlled by positive regulators (ethylene, ABA, and jasmonic acid) and negative regulators (auxin and gibberellin) [46, 47]. We found different signal responses among tissue types in terms of abscission induction by cold, but the gene expression data showed no clear pattern. Overall, the initial key activator for the cold-inducible abscission signal is ABA, as indicated by its amplified biosynthesis after cold stress (Fig 3a). ABA, as a cold-inducible hormone [11], mediates early abscission induction of apple fruitlet [48]. It is, thus, no wonder that ABA-dependent signaling may contribute to abscission induction of early developing fruit as a result of cold response.

Though ABA biosynthesis (*MdNCED1*) was upregulated in the early stage, it was soon inhibited (Fig 3a) and the signals for ethylene biosynthesis (*MdACS1*) and reception (*MdETR2*) significantly increased in the later stage (Fig 3e). This may imply that the change from ABA to ethylene signaling in the pedicel explains the abscission induction signals and the acquired competence of AZ cells to respond to ethylene signal during AZ development [49]. After cold stress, the expressions of both GA biosynthesis (*MdGA20ox*) and polar auxin transport (*MdPIN1*) genes were downregulated in fruit, but not in branch and pedicle tissues. We believe that this downregulation may also contribute to signals of AZ formation by potentially increasing the sensitivity to ethylene in the pedicel, considering that the auxin signal in fruit affects ethylene sensitivity of the AZ [1]. Finally, a more pronounced changes in gene expression after cold stress were present in branch tissue than in the other two tissues with regard to AZ formation and cold response. Although all tissues might potentially respond to cold stress, the branch tissue maintained the upregulation of downstream cold responses. After 18 hr since the cold shock, the expressions of *MdCBF2* and *MdLAC7* were the highest in branch tissues. The expression of these genes increased significantly from 6 to 18 hr in the cold-stressed branch compared with that in the control.

### Exogenous ABA application impacted the responses for abscission induction but in a different manner from that of cold response

Our *ex vivo* experiments with apple subunits demonstrated that cold stress induced ABA-dependent signals, especially in branch and pedicel tissues. Although previous studies have shown that ABA biosynthesis and signaling are controlled by environmental stress signals such as cold or drought [50, 51], it is worth noting that the response pattern of the ABA transporter quite differed between cold and exogenous ABA treatments. In particular, *MdABCG25* expression was significantly upregulated in branch and pedicel tissues under cold stress, while it was downregulated in treatments with exogenous ABA (Fig 4b). This may be attributed to the ABA homeostasis through its negative feedback regulation [52, 53]. In terms of ethylene biosynthesis, cold stress contributed to increase *MdACS1* expression earlier in the pedicel, whereas exogenous ABA application resulted in a delayed upregulation of this gene in branch and fruit tissues. In contrast, the AZ development intensified when apple subunits were treated with combined cold stress and exogenous ABA (S4 Fig). These results imply that cold-induced signals leading to abscission induction may occur in a different manner from those typically amplified by ABA itself.

Furthermore, the correlation analysis of qRT-PCR genes indicated that, under cold stress, the expression of *MdACS1* was consistently positively correlated to *MdLAC7* expression, which mediates lignin polymerization in the branch and pedicel, regardless of exogenous ABA (S5 Fig). In branch and pedicel tissues exposed to cold stress but no exogenous ABA, there was a significant correlation between the cold response gene *MdCS120-like* and other genes such as *MdABCG25* and *MdLAC7*—*MdCS120-like* was positively correlated with *MdABCG25* and negatively correlated with *MdLAC7*. In the cold-stressed fruit tissue, there was no significant relationship between *MdCS120-like* and *MdABCG25*, while the same genes were negatively correlated in fruit tissue subjected to treatments with exogenous ABA and the control. These particular patterns of gene correlation may reflect the characteristics of cold-inducible ABA-dependent signals leading to abscission induction.

**Fig 5:**
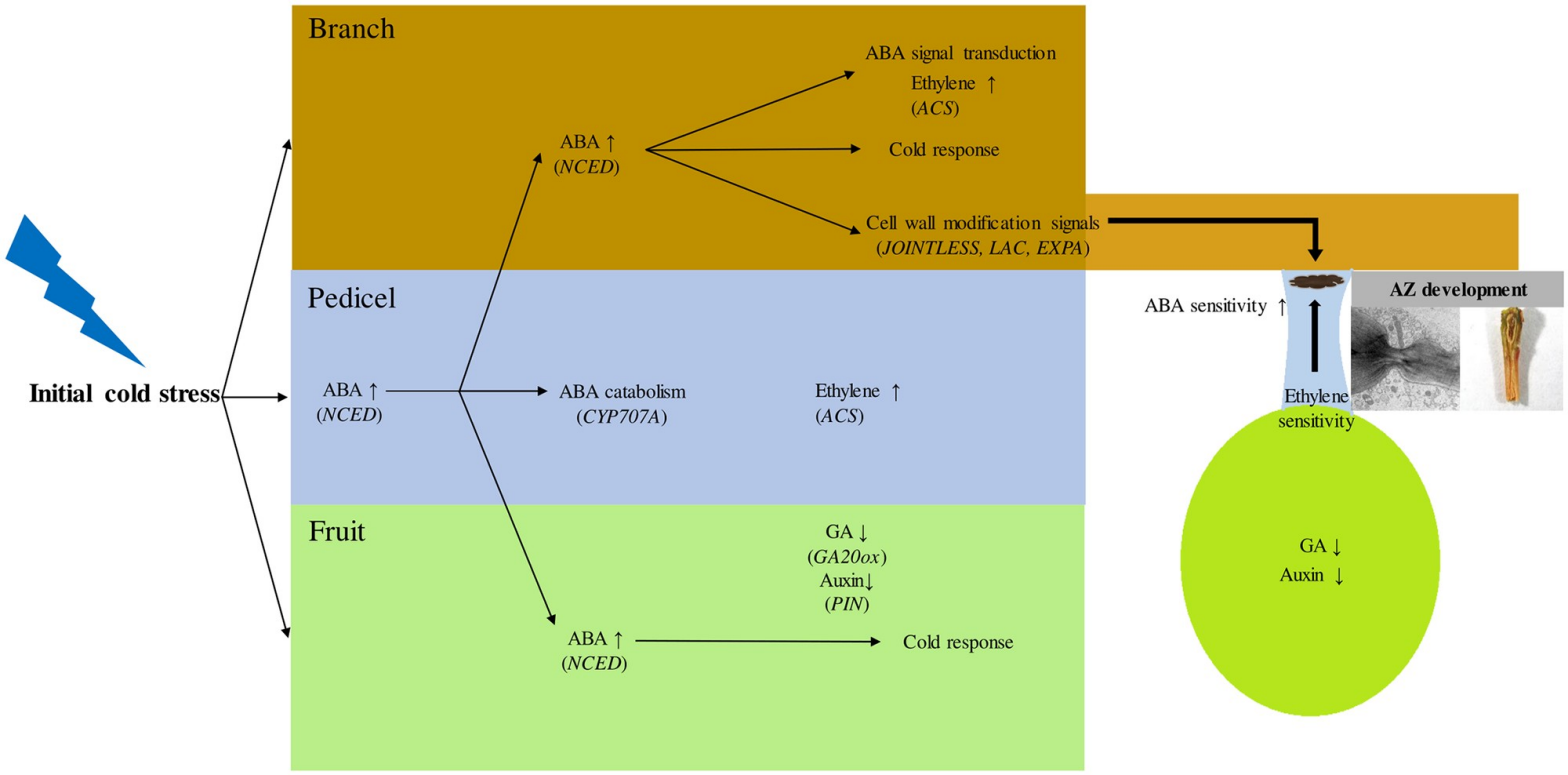
A scheme of the abscission signaling induced by cold stress in early developing apple.

On the basis of our results, we propose a hypothetical scheme for early induction of fruit abscission with spatial signals induced by cold stress (Fig 5). As a primary cold response, ABA biosynthesis is promoted first in the pedicel and its biosynthesis is activated in the neighboring branch and pedicel tissues, possibly as a consequence of positive feedback, concurrent with the upregulation of the ABA transporter. The cold-induced drought responses also appear to contribute to the upregulation of abscission signals. The signals for AZ formation are stimulated largely from the branch rather than the pedicel, in terms of cell wall modification. Fruit coordinates the abscission induction through increase in ethylene sensitivity in the pedicel by inhibiting auxin flow and gibberellin signals. The molecular mechanisms of cold-induced abscission process under ABA inhibition remain to be further elucidated.

## Conclusions

Premature fruit drop is often associated with abiotic stresses. During the early development, apple fruit are exposed to abnormal cold conditions following temperature fluctuations. Our results indicate that apple trees having early developing fruit produce signals for abscission induction in response to cold. Once the initial elevation of ABA biosynthesis in the pedicel spreads to adjacent organs, increased ABA signaling orchestrates cold response. AZ development is coordinated with the signals for cell wall modification produced in the branch and regulation of hormones in the fruit. Based on these findings, we suggest that ABA-dependent responses to cold contribute to abscission induction signaling in early developing apple.

## Supporting information

S1 Fig Early developing fruit collected from a six-year-old Hongro/M9 apple tree in May 2018. (a) 3 cm-sized fruit undergoing abscission. (b) Control.
(PDF)

S2 Fig Transcriptome profile of Hongro/M9 young apple fruit/pedicel mixed tissues undergoing abscission. (a) 439 DEGs filtered by a criteria of |log_2_ fold change| > 1 and FDR value < 0.05. Of those, 324 were upregulated and 115 were downregulated. (b) Gene ontology list with a criteria of *p* < 0.05. (c) A list of selected DEGs of Hongro/M9 young apple fruit/pedicel mixed tissues undergoing abscission. The selected 92 DEGs were categorized into nine groups: cell wall modification, oxidation-reduction, senescence, DNA binding, phytohormone signal transduction, dehydration, degradation, phosphorylation, and hydrolysis.
(PDF)

S3 Fig The excised subunits collected from a six-year-old Hongro/M9 apple tree in May 2019. (a) Apple subunit consisting of branch, pedicel, and fruit. (b) Apple subunits exposed to 2 hr of *ex vivo* cold shock were severely damaged and more dehydrated after 168 hr of incubation at 25 °C compared to the control.
(PDF)

S4 Fig Transmission electron microscopic images of the abscission zone (AZ) cortical cells at proximal tissues in pedicel. Samples were grouped by treatments as follows: Control (water); ABA (125 mg/L of ABA); Cold (initial cold shock at 4 °C for 2 hr); Cold + ABA (125 mg/L ABA followed by initial cold shock at 4 °C for 2 hr). AZ cortical cells were observed with a LIBRA 120 transmission electron microscope at an acceleration voltage of 120 kV, magnification with 4 k (left) and 6.3 k (right). Arrows indicate the development of cytoplasmic vesicles.
(PDF)

S5 Fig Correlation matrix for qRT-PCR expressions of target genes shown in Fig 4. Correlation coefficient *r* was calculated between the relative expression levels of genes from all time points. * indicate significant difference at *p* < 0.05 by Student’s *t*-test.
(PDF)

S1 Table List of primers used for qRT-PCR in this study. (PDF)

S1 Text Material and methods. (DOCX)
