# Supplementary material for: Cold stress triggers premature fruit abscission through ABA-dependent signal transduction in early developing apple
Source: PLoS One. 2021 Apr 9;16(4):e0249975. doi: 10.1371/journal.pone.0249975 (PMC8034736; doi:10.1371/journal.pone.0249975)
Supplement: S1 Fig — (a) 3 cm-sized fruit undergoing abscission. (b) Control. (PDF) [file pone.0249975.s001.pdf]

a b

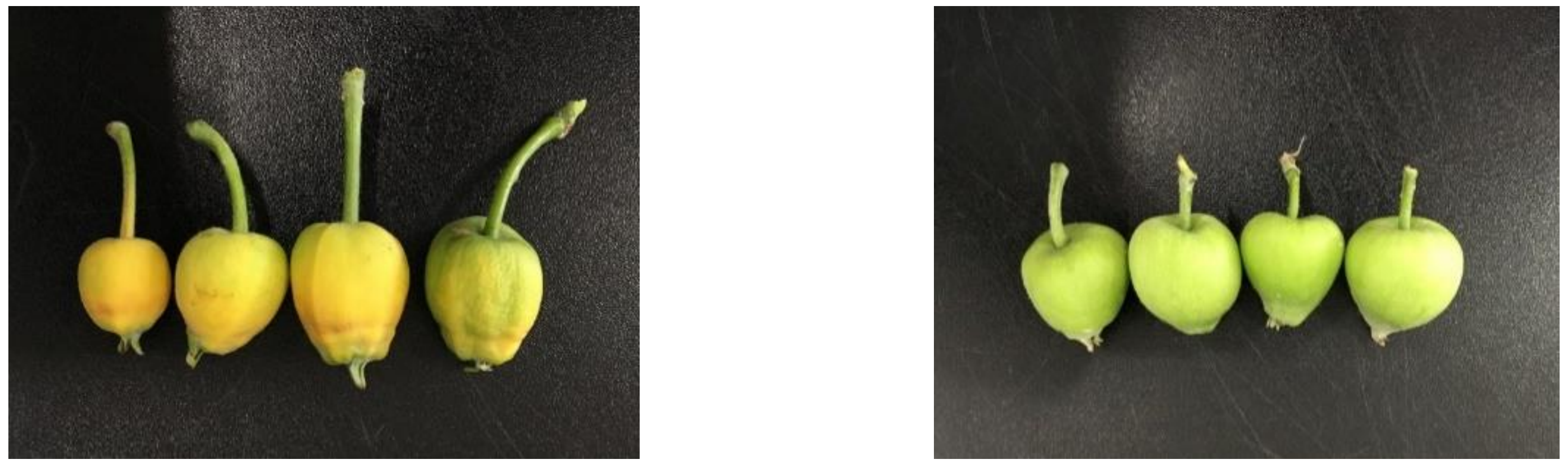

S1 Fig. Early developing fruit collected from a six-year-old Hongro/M9 apple tree in May 2018. (a) 3 cm-sized fruit undergoing abscission. (b) Control.
