# Supplementary material for: Cold stress triggers premature fruit abscission through ABA-dependent signal transduction in early developing apple
Source: PLoS One. 2021 Apr 9;16(4):e0249975. doi: 10.1371/journal.pone.0249975 (PMC8034736; doi:10.1371/journal.pone.0249975)
Supplement: S2 Fig — (a) 439 DEGs filtered by a criteria of |log2 fold change| > 1 and FDR value < 0.05. Of those, 324 were upregulated and 115 were downregulated. (b) Gene ontology list with a criteria of p < 0.05. (c) A list of selected DEGs of Hongro/M9 young apple fruit/pedicel mixed tissues undergoing abscission. The selected 92 DEGs were categorized into nine groups: cell wall modification, oxidation-reduction, senescence, DNA binding, phytohormone signal transduction, dehydration, degradation, phosphorylation, and hydrolysis. (PDF) [file pone.0249975.s002.pdf]

(a)

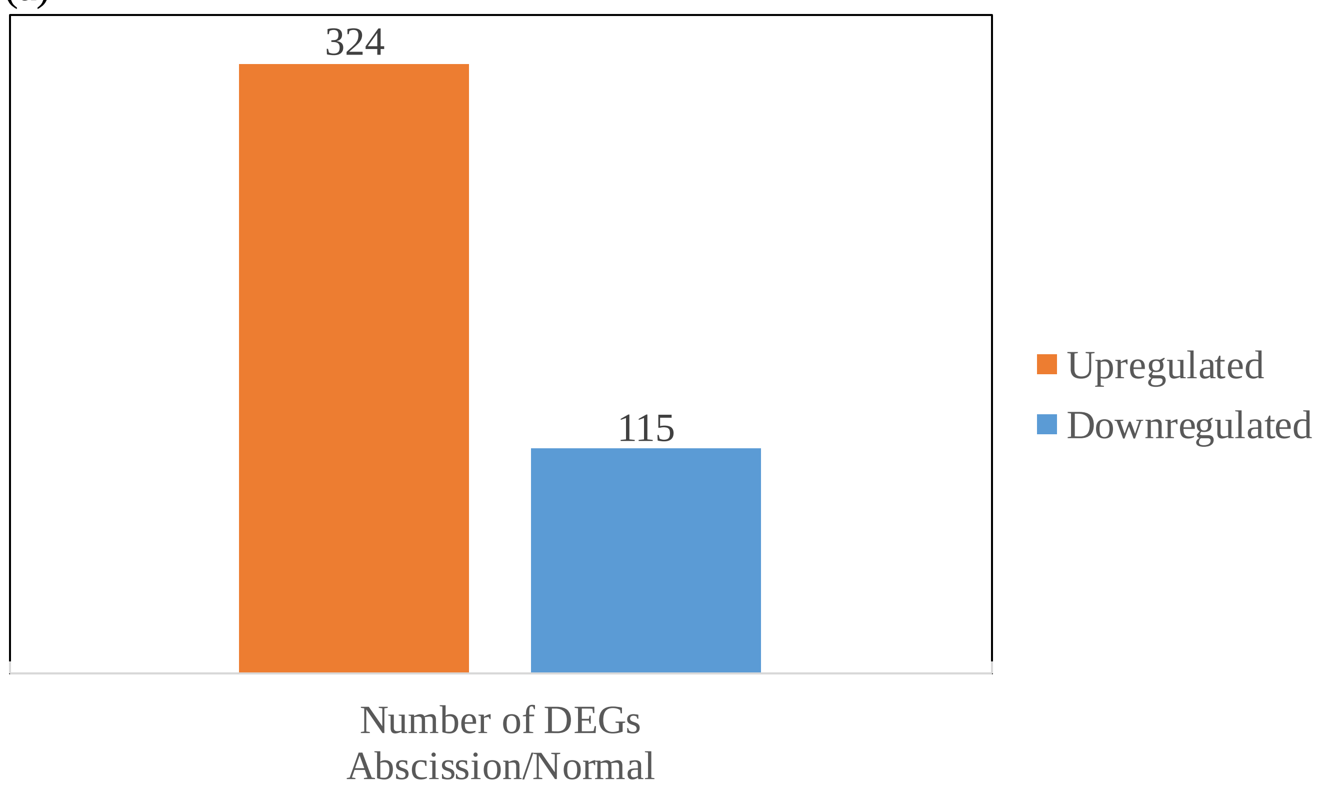

(b)

Gene Ontology Term	DEG count	p-value
protein binding (GO:0005515)	6	0.0001
iron ion binding (GO:0005506)	5	0.0002
response to stress (GO:0006950)	3	0.0008
oxidoreductase activity, acting on paired donors, with incorporation or reduction of molecular oxygen (GO:0016705)	2	0.0011
oxidation-reduction process (GO:0055114)	11	0.0016
flavin adenine dinucleotide binding (GO:0050660)	3	0.0028
oxidoreductase activity (GO:0016491)	4	0.0113
defense response (GO:0006952)	2	0.0144
metabolic process (GO:0008152)	3	0.0222
copper ion binding (GO:0005507)	2	0.0346
hydrolase activity (GO:0016787)	2	0.0424

(c)

Classification	Gene ID	InterPro ID	Blastp annotation (Swiss-Prot)	E-value	Annotation	log2FC (Abscission/Normal)	FDR
**Cell wall modification**	MD08G1195600	IPR012334	Probable pectinesterase/pectinesterase inhibitor 41	0	Pectin lyase fold	-15.074	0.004
	MD07G1011600	IPR011050	Polygalacturonase	3.00E-175	Pectin lyase fold/virulence factor	-9.969	0.036
	MD10G1179100	IPR011050	Polygalacturonase	0	Pectin lyase fold/virulence factor	-9.927	0.006
	MD04G1008100	IPR011050	Probable pectinesterase 29	4.00E-136	Pectin lyase fold/virulence factor	-6.562	0.046
	MD02G1038100	IPR007112	EG45-like domain containing protein	2.00E-14		-4.510	0.034
	MD05G1341800	IPR012334	Pectinesterase/pectinesterase inhibitor	0	Pectin lyase fold	4.363	0.035
	MD06G1195100	IPR002963	Expansin-A1	8.00E-153		7.079	0.005
	MD00G1118800	IPR000620	WAT1-related protein At3g30340	7.00E-124		7.649	0.001
	MD12G1210600	IPR012334	Putative pectinesterase/pectinesterase inhibitor 22	0	Pectin lyase fold	9.221	0.009
	MD02G1142600	IPR012334	Pectinesterase PPME1	0.0003	Pectin lyase fold	10.852	0.001
	MD02G1104500	IPR011050	Probable pectinesterase/pectinesterase inhibitor 12	0	Pectin lyase fold/virulence factor	12.188	0.001
**Oxidation-reduction**	MD10G1298700	IPR016213	Polyphenol oxidase, chloroplastic	0	Polyphenol oxidase	-13.964	0.005
	MD02G1139900	IPR002355	Dehydrin Xero 1	2.00E-20	Multicopper oxidase, copper-binding site	-13.345	0.005
	MD10G1085800	IPR002403	Allene oxide synthase 3	0		-12.995	0.008
	MD15G1306400	IPR002355	Transcription factor bHLH93	9.00E-91	Multicopper oxidase, copper-binding site	-12.957	0.008
	MD05G1266200	IPR006094	Berberine bridge enzyme-like 8	0		-11.356	0.006
	MD05G1302400	IPR011598	Transcription factor bHLH51	6.00E-34		-10.773	0.012
	MD03G1082200	IPR002213	UDP-glucose iridoid glucosyltransferase	4.00E-161	UDP-glucuronosyl/UDP-glucosyltransferase	-9.674	0.006
	MD02G1248200	IPR002213	Beta-D-glucosyl crocetin beta-1,6-glucosyltransferase	2.00E-159	UDP-glucuronosyl/UDP-glucosyltransferase	-9.648	0.027
	MD06G1089200	IPR006694	Beta-carotene 3-hydroxylase 1, chloroplastic	2.00E-131		-9.326	0.018
	MD10G1299300	IPR016213	Polyphenol oxidase, chloroplastic	0	Polyphenol oxidase	-8.960	0.025
	MD04G1142900	IPR002355	Laccase-7	0	Multicopper oxidase, copper-binding site	-8.786	0.046
	MD06G1162600	IPR002401	Cytochrome P450 71A9	0	Cytochrome P450, E-class, group I	-8.614	0.032
	MD10G1111300	IPR002213	UDP-glycosyltransferase 74E2	4.00E-155	UDP-glucuronosyl/UDP-glucosyltransferase	-8.277	0.048
	MD10G1298500	IPR016213	Polyphenol oxidase, chloroplastic	0	Polyphenol oxidase	-7.955	0.040
	MD02G1069300	IPR007828	Inositol oxygenase 2	1.00E-177		-7.849	0.034
	MD01G1195400	IPR002328	Sorbitol dehydrogenase	0		-7.821	0.009
	MD05G1207300	IPR004294	9-cis-epoxycarotenoid dioxygenase NCED1, chloroplastic	0		-7.293	0.020
	MD10G1299400	IPR016213	Polyphenol oxidase, chloroplastic	0	Polyphenol oxidase	-6.890	0.045
	MD10G1138400	IPR002401	Cytochrome P450 87A3	2.00E-146	Cytochrome P450, E-class, group I	-5.223	0.027
	MD15G1074800	IPR002401	Cytochrome P450 81E8	9.00E-166	Cytochrome P450, E-class, group I	-4.373	0.028
	MD06G1213800	IPR002401	Cytochrome P450 CYP736A12	0	Cytochrome P450, E-class, group I	-4.256	0.035
	MD07G1208400	IPR002213	UDP-glycosyltransferase 76F1	4.00E-160	UDP-glucuronosyl/UDP-glucosyltransferase	4.003	0.034
	MD10G1014500	IPR002328	Alcohol dehydrogenase 1	0	Alcohol dehydrogenase, zinc-type, conserved site	4.247	0.034
	MD03G1081900	IPR002213	UDP-glucose iridoid glucosyltransferase	8.00E-161	UDP-glucuronosyl/UDP-glucosyltransferase	4.388	0.032
	MD13G1058300	IPR002401	Cytochrome P450 CYP749A22	0	Cytochrome P450, E-class, group I	4.826	0.048
	MD02G1083600	IPR002213	UDP-glycosyltransferase 91C1	2.00E-136	UDP-glucuronosyl/UDP-glucosyltransferase	4.829	0.031
	MD15G1073500	IPR016040	3-oxo-Delta(4,5)-steroid 5-beta-reductase	0	NAD(P)-binding domain	5.027	0.009
	MD16G1020300	IPR007736	Probable peroxygenase 4	7.00E-92	Caleosin-related	5.497	0.006
	MD17G1179700	IPR007828	Inositol oxygenase	9.00E-86	Inositol oxygenase	5.593	0.021
	MD16G1116200	IPR002401	Geraniol 8-hydroxylase	0	Cytochrome P450, E-class, group I	5.651	0.008
	MD02G1093200	IPR002401	Flavonoid 3'-monooxygenase	4.00E-137	Cytochrome P450, E-class, group I	6.744	0.003
	MD11G1091800	IPR002401	Cytochrome P450 71A6	0	Cytochrome P450, E-class, group I	7.026	0.001
	MD13G1041900	IPR000894	Ribulose bisphosphate carboxylase small chain, chloroplastic	5.00E-23	Ribulose bisphosphate carboxylase small chain, domain	7.358	0.003
	MD15G1353500	IPR012336	Peroxiredoxin-2B	2.00E-16	Thioredoxin-like fold	7.676	0.050
	MD13G1041800	IPR000894	Ribulose bisphosphate carboxylase small chain 1, chloroplastic	2.00E-29	Ribulose bisphosphate carboxylase small chain, domain	8.113	0.004
	MD01G1203800	IPR002401	Cytochrome P450 89A2	5.00E-73	Cytochrome P450, E-class, group I	8.196	0.033
	MD03G1204900	IPR002401	Cytochrome P450 716B1	1.00E-63	Cytochrome P450, E-class, group I	9.004	0.017
	MD15G1267300	IPR002213	Anthocyanidin 3-O-glucosyltransferase 5	0	UDP-glucuronosyl/UDP-glucosyltransferase	6.673	0.031
	MD10G1286900	IPR002213	Anthocyanidin 3-O-glucoside 2"-O-glucosyltransferase	7.00E-26	UDP-glucuronosyl/UDP-glucosyltransferase	8.291	0.034
	MD10G1287000	IPR002213	Anthocyanidin 3-O-glucoside 2"-O-glucosyltransferase	2.00E-129	UDP-glucuronosyl/UDP-glucosyltransferase	10.348	0.005
**Senescence**	MD10G1269600	IPR007608	Phosphatidylethanolamine N-methyltransferase	1.1	Senescence regulator S40	-9.047	0.015
	MD00G1093200	IPR007608	Putative glycylpeptide N-tetradecanoyltransferase	1.5	Senescence regulator S40	-8.182	0.010
	MD01G1072000	IPR004864	Desiccation protectant protein Lea14 homolog	1.00E-27		8.626	0.024
	MD07G1281400	IPR004864	late embryogenesis abundant protein	1.00E-18	Late embryogenesis abundant protein (LEA2) subgroup	8.717	0.025
**DNA binding**	MD10G1198400	IPR003441	NAC domain-containing protein 21/22	2.00E-118	NAC domain	-8.476	0.015
	MD09G1053700	IPR003441	NAC domain-containing protein 100	1.00E-128	NAC domain	-7.911	0.018
	MD13G1069200	IPR003441	NAC transcription factor 29	1.00E-110	NAC domain	-6.887	0.020
	MD17G1001400	IPR004333	Squamosa promoter-binding-like (SBP) protein 13B	5.00E-39	SBP domain	4.168	0.018
	MD15G1306400	IPR011598	Transcription factor basic helix-loop-helix (bHLH) 93	9.00E-91	Myc-type, basic helix-loop-helix (bHLH) domain	4.178	0.046
	MD06G1120100	IPR025610	Transcription factor basic helix-loop-helix (bHLH) 14	1.00E-60	Transcription factor MYC/MYB N-terminal	4.909	0.029
	MD04G1046000	IPR005202	Protein SHORT-ROOT (SHR)	5.00E-143	Transcription factor GAI, RGA, SCR (GRAS) family	5.155	0.026
	MD06G1031700	IPR003441	NAC domain-containing protein 71	3.00E-103	NAC domain	5.256	0.011
	MD15G1407200	IPR011598	Transcription factor basic helix-loop-helix (bHLH) 80	5.00E-68	Myc-type, basic helix-loop-helix (bHLH) domain	5.524	0.025
	MD04G1208700	IPR011598	Transcription factor basic helix-loop-helix (bHLH) 96	6.00E-77	Myc-type, basic helix-loop-helix (bHLH) domain	7.324	0.001
	MD04G1161800	IPR011598	Transcription factor basic helix-loop-helix (bHLH) 140	3.00E-30	Myc-type, basic helix-loop-helix (bHLH) domain	7.803	0.047
**Phytohormone signal transcduction**	MD02G1084600	IPR015655	Protein phosphatase 2C 7	5.00E-145	Protein phosphatase 2C family	-5.838	0.020
	MD15G1082600	IPR002401	Abscisic acid 8'-hydroxylase 4	0	Cytochrome P450, E-class, group I	5.174	0.029
	MD15G1250900	IPR008496	Protein Reversio-to-Ethylene Perception 1 (RTE1)-HOMOLOG	2.00E-102	Reversion-to-Ethylene Perception 1 (RTE1)	8.258	0.031
	MD10G1029100	IPR003496	Abscisic stress-ripening protein 2	1.00E-13	Abscisic acid (ABA)/water deficit stress (WDS) induced protein	10.194	0.006
**Dehydration**	MD02G1139900	IPR000167	Dehydrin Xero 1 (XERO1)	2.00E-20	Dehydrin	-5.946	0.039
	MD02G1140100	IPR000167	Dehydrin 1 (DHN1)	2.00E-14	Dehydrin	3.754	0.021
	MD00G1127700	IPR004926	Late embryogenis abundant protein 41	4.00E-20	Late embryogenesis abundant protein, LEA_3 subgroup	6.858	0.003
**Degradation**	MD08G1068800	IPR002068	18.8 kDa class II heat shock protein	2.00E-60	Hsp20 domain	3.801	0.046
	MD12G1048700	IPR024729	Ubiquitin carboxyl-terminal hydrolase 13	0	Ubiquitin carboxyl-terminal hydrolase 7, ICP0-binding domain	4.090	0.046
	MD05G1183400	IPR002068	22.7 kDa class IV heat shock protein	9.00E-75	Hsp20 domain	4.440	0.049
	MD17G1020300	IPR002068	17.5 kDa class I heat shock protein	2.00E-46	Hsp20 domain	6.294	0.022
	MD12G1113200	IPR019190	Exonuclease V, chloroplastic	5.00E-31	Exonuclease V	7.155	0.049
	MD07G1298000	IPR002068	Small heat shock protein, chloroplastic	9.00E-96	Alpha crystallin/Hsp20 domain	8.219	0.006
**Phosphorylation**	MD08G1073100	IPR020635	Serine/threonine-protein kinase RIPK	6.00E-173	Tyrosine-protein kinase, catalytic domain	-8.348	0.027
	MD13G1153400	IPR003661	Phytochrome B	0	Signal transduction histidine kinase, dimerisation/phosphoacceptor domain	4.220	0.031
	MD07G1059500	IPR000387	Uncharacterized protein YnbD	9.00E-12	Tyrosine specific protein phosphatases domain	5.297	0.024
	MD04G1172700	IPR020635	Probable serine/threonine-protein kinase WNK5	0	Tyrosine-protein kinase, catalytic domain	5.508	0.010
	MD05G1054300	IPR000719	Dual specificity protein kinase shkC	7.00E-13	Protein kinase domain	5.683	0.007
	MD05G1054200	IPR011009	CBL-interacting serine/threonine-protein kinase 11	0.052	Protein kinase-like domain superfamily	5.826	0.015
	MD02G1021900	IPR011009	Calmodulin-binding receptor-like cytoplasmic kinase 3	2.00E-12	Protein kinase-like domain superfamily	5.905	0.010
	MD05G1031900	IPR011009	Putative cysteine-rich receptor-like protein kinase 35	4.00E-37	Protein kinase-like domain superfamily	8.183	0.018
	MD17G1084100	IPR000719	G-type lectin S-receptor-like serine/threonine-protein kinase CES101	1.00E-12	Protein kinase domain	9.252	0.013
	MD17G1084200	IPR000719	Cysteine-rich receptor-like protein kinase 10	5.00E-45	Protein kinase domain	9.554	0.010
	MD10G1288400	IPR020635	Serine/threonine-protein kinase-like protein ACR4	2.00E-50	Tyrosine-protein kinase, catalytic domain	9.972	0.007
**Hydrolysis**	MD17G1007000	IPR015341	Probable alpha-mannosidase	0	Glycoside hydrolase family 38, central domain	6.682	0.002
	MD12G1221300	IPR000120	Amidase 1	7.00E-43	Amidase	6.792	0.016

S2 Fig. Transcriptome profile of Hongro/M9 young apple fruit/pedicel mixed tissues undergoing abscission. (a) 439 DEGs filtered by a criteria of |log2 fold change| > 1 and FDR value < 0.05. Of those, 324 were upregulated and 115 were downregulated. (b) Gene ontology list with a criteria of $p < 0.05$. (c) A list of selected DEGs of Hongro/M9 young apple fruit/pedicel mixed tissues undergoing abscission. The selected 92 DEGs were categorized into nine groups: cell wall modification, oxidation-reduction, senescence, DNA binding, phytohormone signal transduction, dehydration, degradation, phosphorylation, and hydrolysis.
