# Supplementary material for: Cold stress triggers premature fruit abscission through ABA-dependent signal transduction in early developing apple
Source: PLoS One. 2021 Apr 9;16(4):e0249975. doi: 10.1371/journal.pone.0249975 (PMC8034736; doi:10.1371/journal.pone.0249975)
Supplement: S3 Fig — (a) Apple subunit consisting of branch, pedicel, and fruit. (b) Apple subunits exposed to 2 hr of ex vivo cold shock were severely damaged and more dehydrated after 168 hr of incubation at 25 °C compared to the control. (PDF) [file pone.0249975.s003.pdf]

(a) (b)

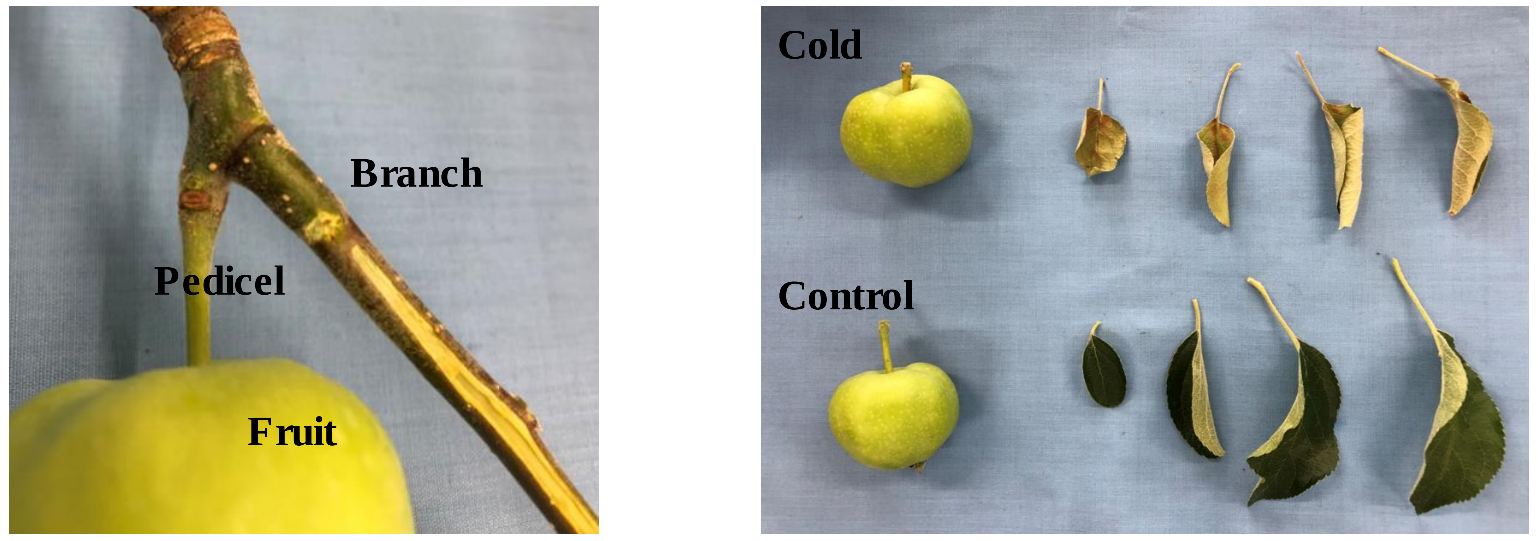


**S3 Fig. The excised subunits collected from a six-year-old Hongro/M9 apple tree in May 2019.** (a) Apple subunit consisting of branch, pedicel, and fruit. (b) Apple subunits exposed to 2 hr of ex vivo cold shock were severely damaged and more dehydrated after 168 hr of incubation at 25 °C compared to the control.
