# Supplementary material for: Cold stress triggers premature fruit abscission through ABA-dependent signal transduction in early developing apple
Source: PLoS One. 2021 Apr 9;16(4):e0249975. doi: 10.1371/journal.pone.0249975 (PMC8034736; doi:10.1371/journal.pone.0249975)
Supplement: S4 Fig — Samples were grouped by treatments as follows: Control (water); ABA (125 mg/L of ABA); Cold (initial cold shock at 4 °C for 2 hr); Cold + ABA (125 mg/L ABA followed by initial cold shock at 4 °C for 2 hr). AZ cortical cells were observed with a LIBRA 120 transmission electron microscope at an acceleration voltage of 120 kV, magnification with 4 k (left) and 6.3 k (right). Arrows indicate the development of cytoplasmic vesicles. (PDF) [file pone.0249975.s004.pdf]

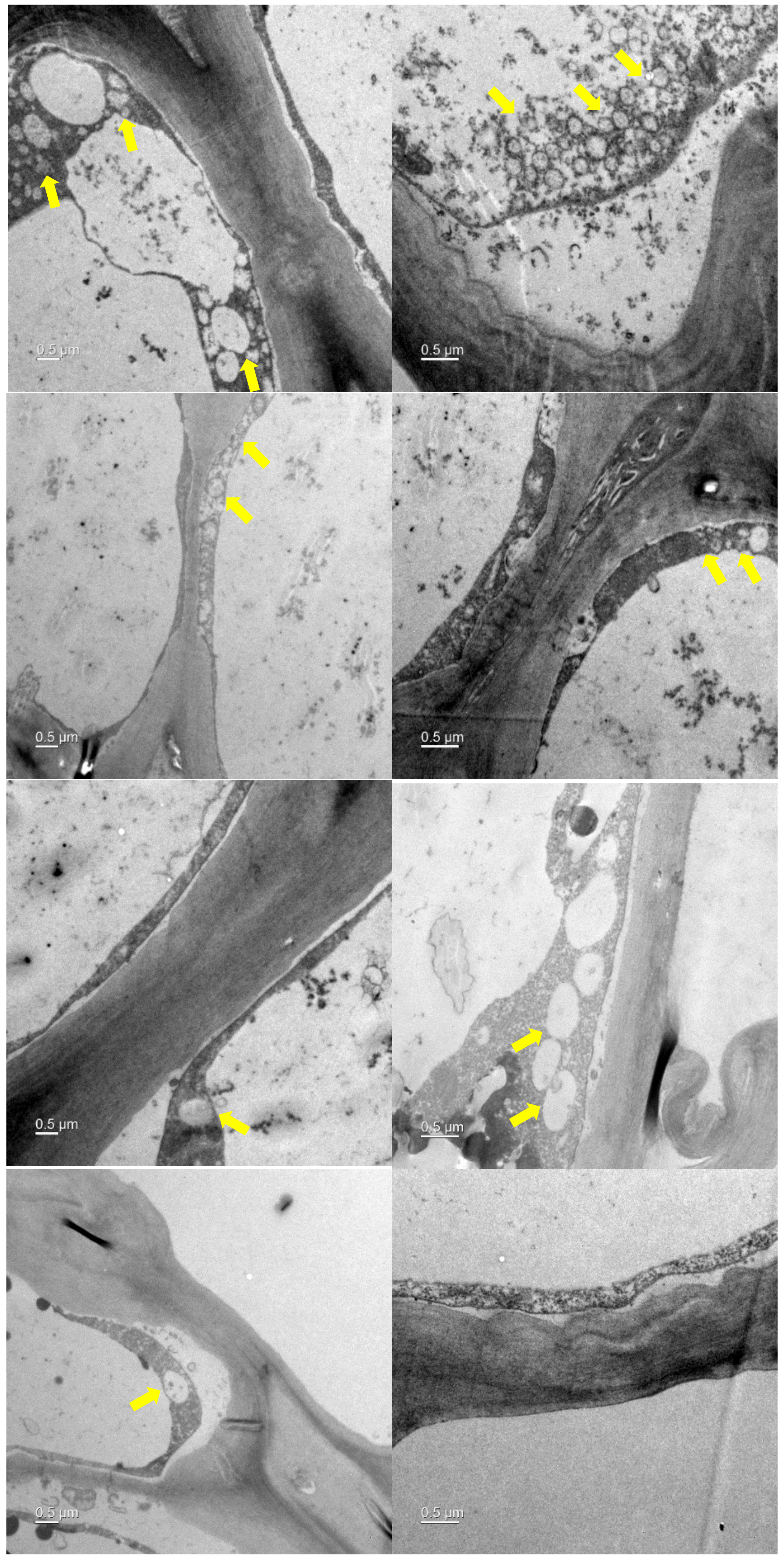

S4 Fig. Transmission electron microscopic images of the abscission zone (AZ) cortical cells at proximal tissues in pedicel. Samples were grouped by treatments as follows: Control (water); ABA (125 mg/L of ABA); Cold (initial cold shock at 4 °C for 2 hr); Cold + ABA (125 mg/L ABA followed by initial cold shock at 4 °C for 2 hr). AZ cortical cells were observed with a LIBRA 120 transmission electron microscope at an acceleration voltage of 120 kV, magnification with 4 k (left) and 6.3 k (right). Arrows indicate the development of cytoplasmic vesicles.
