# Supplementary material for: Cold stress triggers premature fruit abscission through ABA-dependent signal transduction in early developing apple
Source: PLoS One. 2021 Apr 9;16(4):e0249975. doi: 10.1371/journal.pone.0249975 (PMC8034736; doi:10.1371/journal.pone.0249975)
Supplement: S5 Fig — Correlation coefficient r was calculated between the relative expression levels of genes from all time points. * indicate significant difference at p < 0.05 by Student’s t-test. (PDF) [file pone.0249975.s005.pdf]

Cold + ABA

Fruit

	MdCS120-like	*MdABCG25*	*MdACS4*
MdABCG25	0.984		
MdACS4	-0.363	-0.431 *	
MdLAC7	-	-	-

Branch

	MdCS120-like	*MdABCG25*	*MdACS1*
MdABCG25	0.973		
MdACS1	-0.870	-0.957 *	
MdLAC7	-0.975	-0.916	0.761 *

Pedicel

	MdCS120-like	*MdABCG25*	*MdACS1*
MdABCG25	0.840		
MdACS1	-0.326	0.210 *	
MdLAC7	-0.463	0.038	0.723 *

Cold

Fruit

	MdCS120-like	*MdABCG25*	*MdACS1*
MdABCG25	0.996		
MdACS1	0.195	0.269	
MdLAC7	-	-	-

Branch

	MdCS120-like	*MdABCG25*	*MdACS1*
MdABCG25	0.046 *		
MdACS1	-0.576	0.775	
MdLAC7	-0.023 *	0.974	0.761 *

Pedicel

	MdCS120-like	*MdABCG25*	*MdACS1*
MdABCG25	0.532 *		
MdACS1	-0.967	-0.504 *	
MdLAC7	-0.782 *	-0.508	0.599 *

ABA

Fruit

	MdCS120-like	*MdABCG25*	*MdACS1*
MdABCG25	-0.647 *		
MdACS1	0.096	0.569	
MdLAC7	-	-	-

Branch

	MdCS120-like	*MdABCG25*	*MdACS1*
MdABCG25	-0.223		
MdACS1	0.539	0.166 *	
MdLAC7	0.757	0.252	0.922 *

Pedicel

	MdCS120-like	*MdABCG25*	*MdACS1*
MdABCG25	-0.686		
MdACS1	0.354	0.169	
MdLAC7	0.710 *	-0.372	0.852

Control

Fruit

	MdCS120-like	*MdABCG25*	*MdACS1*
MdABCG25	-0.972 *		
MdACS1	-0.867	0.946 *	
MdLAC7	-	-	-

Branch

	MdCS120-like	*MdABCG25*	*MdACS1*
MdABCG25	0.128		
MdACS1	-0.373	-0.861	
MdLAC7	0.747 *	0.728	-0.882 *

Pedicel

	MdCS120-like	*MdABCG25*	*MdACS1*
MdABCG25	0.136 *		
MdACS1	-0.387	0.761 *	
MdLAC7	-0.532	0.435	0.910

S5 Fig. Correlation matrix for qRT-PCR expressions of target genes shown in Fig 4. Correlation coefficient r was calculated between the relative expression levels of genes from all time points. * indicate significant difference at $p < 0.05$ by Student's t-test.
