# Supplementary material for: Cold stress triggers premature fruit abscission through ABA-dependent signal transduction in early developing apple
Source: PLoS One. 2021 Apr 9;16(4):e0249975. doi: 10.1371/journal.pone.0249975 (PMC8034736; doi:10.1371/journal.pone.0249975)
Supplement: S1 Table — (PDF) [file pone.0249975.s006.pdf]

S1 Table. List of primers used for qRT-PCR in this study.

Gene name	Gene accession	Forward (5' to 3')	Reverse (5' to 3')
MdNCED1	XM_008384748	GTCCATGCCCTCCAGTTCAA	TTTGGGAAATAGGGGACGGC
MdCYP707A	XM_008374924	CTGTTTGCAGCCCAAGACAC	GGTCTTTTGCTCAGCCTCCA
MdPYL8	XM_008382402	GCGTTTTGATTGAGGAAGATGA	TGTGGTGCCTCCGAATGTAG
MdPYL3	XM_008377890	TATCGGAAAATGAGCATCGGCG	CACCAAGGACCAGACGAGA
MdPP2C	XM_029090720	ATTTTACGTAATGGCAAGTGATGGA	AGTCGCCAATCTCTTGGAGC
MdWRKY40	XM_008394247	TCAAGGCTAAGATTTCAAGGGCT	TCTTTGAACCTTCTTTTTGACAGG
MdABI5	XM_008385717	CGAGCAAGGAAGCAGGCATA	CACTGGGCAACACCTTCTCT
MdABCG25	XM_008382769	ACATATACAGTAGCAGCAGAGATG	ACACACGTCCACGAACTTGA
MdCBF2/DREB1A	XM_029104167	CTCTGACGCAGCACAACAAA	CCAGCTGGCTTCGAGTCAA
MdCBF3/DREB1E	XM_029104587	TTCGAAGGCAGCATTACTCCA	CTTTGAGTGGGACCCAGGAC
MdCS120-like	NM_001328720	GGCGGCCACAAGTAGAT	TGGACGACTGCGTATCACAC
MdRD22	XM_017333810	TCCAGCCTGCAGATTTCACA	TCCCAACATCGACATGGGTG
MdWRKY57	XM_008391196	CACCGTCCGAGATACCGTAA	ACCATATTTCCGCCAACGGT
MdJb	NM_001328882	TCAACGATCTTCAAAGAAATGCGA	ACCCTCCTCCGTAATCAGGT
MdEXPA10	XM_008343364	ATGCATGGTCCCAACAGCTC	GCCATTTTCAGTGAGGAAATGCT
MdLAC7	XM_008342340	CCATATGGCACGGTTAGCCT	TGTTCACACTGAACGAGTGCT
MdACS1	XM_008344016	CTTTTCTCTCTCTATCTGAGTCGCA	GCTGCCGCTGCTCAATATCT
MdETR2	NM_001328941	CAGAGGTCACGATTCCAGCA	CCTTGCATCATCTGCACTAGC
MdERF1	NM_001300880	GCGGAGGACCTCTGGTCA	TCATCACTTGTCACTACTTTGGTG
MdPIN1	XM_008395204	TGATATTCTCAGCACAGGGGTT	TGAGTCCCATTTTCTGAAGCAC
MdIAA9	MD08G1207300[a]	AGATGCCATCGGCCTAGGTG	CGTAGGGGAAAGTGAAAGGCT
MdIAA20	MD04G1225000[a]	ACCACTGCAATCCGACATGA	TGCGACAAGATTGCTTACTTGG
MdGA20ox	XM_008378277	ATAAGAACGGCCCTGGCTTT	GGAAGGAGGGTTTGGGTTCC
MdRGL2	NM_001328987	ACGTCATCGATTTCGGGCTT	AGAACGTCGGTGTTATCGGG
MDP0000336547	MDP0000336547[a]	TACTGGAAGACCCGTTTTGG	CTTCCTCGTCGTCATCGAAT

[a] Genes were selected and annotated from GDR database (www.rosaceae.org)
