# Supplementary material for: Cold stress triggers premature fruit abscission through ABA-dependent signal transduction in early developing apple
Source: PLoS One. 2021 Apr 9;16(4):e0249975. doi: 10.1371/journal.pone.0249975 (PMC8034736; doi:10.1371/journal.pone.0249975)
Supplement: S1 Text — (DOCX) [file pone.0249975.s007.docx]

**S1 Text. Material and Methods**

**RNA sequencing and bioinformatics analyses**

RNA integrity number (RIN) of each sample was measured using Agilent 2100 bioanalyzer (Palo Alto, CA, USA) and samples with RIN 7.5 or above were sent to CnK genomics for sequencing. Libraries were prepared using the Illumina TruSeq Stranded mRNA sample preparation kit (San Diego, CA, USA), and a total of six libraries were constructed using an Illumina Nexseq 500 platform (San Diego, CA, USA). Raw reads were trimmed by filtering out adaptors with a minimum length of 75 bp using Trimmomatic v0.36 (http://www.usadellab.org/cms/index.php?page=trimmomatic) [1]. The quality score was evaluated using fastqc (http://www.bioinformatics.babraham.ac.uk/projects/fastqc) both before and after trimming. Trimmed reads were aligned to apple reference genome GDDH13 v1.1 (http://www.iris.angers.inra.fr/gddh13) using HISAT2 software [2]. Across all the libraries, gene counts were calculated for each predicted coding DNA sequence using FeatureCounts v1.5.2. Read counts in the range of 21,543,441 to 24,876,113 were obtained with Q30 ratio > 0.9. Reads per kilobase per million (RPKM) values were counted from BAM files. Differentially expressed genes (DEGs) with a false discovery rate (FDR) value of < 0.05 and |log2 fold change| > 1 were selected using EdgeR Bioconductor software (Empirical analysis of digital gene expression data in R) [3].

Functional enrichment analysis of DEGs was performed using InterPro2 (http://www.ebi.ac.uk/interpro) and SwissProt ([www.ebi.ac.uk/swissprot](http://www.ebi.ac.uk/swissprot)). We identified 439 DEGs using both a FDR value < 0.05 and |log2 fold change| > 1 as cut-offs. 324 genes were up-regulated, and 115 genes were down-regulated (S1a Fig). The major gene ontology (GO) classification included the stress response (GO:0006950), process of oxidation-reduction (GO:0055114, GO:0016705, and GO:0016491), protein binding (GO:005515), and flavin adenine dinucleotide binding (GO:0050660) (S1b Fig). Among 439 DEGs, 269 genes were annotated to the InterPro2 database for the functional enrichment analysis.

**UPLC-QTOF-MS analysis**

Each 0.05 g ground sample was extracted with a 1 mL of ethanol: water: formic acid (80:20:1) solution and incubated for more than 12 hr at 4 °C. The extracted samples were centrifuged at 15,000 *g* for 5 min. Supernatants were diluted 10-fold with 100 % methanol and analysed in both positive and negative ionization modes. The UPLC-QTOF-MS system consisted of Thermo Scientific Dionex Ultimate 3000 (Sunnyvale, CA, USA) and AB SCIEX Triple TOF 5600 system (Framingham, MA, USA). The analytical column was a U-VDSpher PUR 100 C18-E (2.1 mm x 100 mm, 1.8 μm) column (VDS optilab, Germany) and the solvent composition was A=0.1% formic acid in water, B=0.1% formic acid in acetonitrile. The solvent gradient was: 100 % A 0 % B, 0-10 min; linear gradient to 80 % A 20 % B, 10-12 min; linear gradient to 70 % A 30 % B, 12-15 min; the composition held at 5 % A 95 % B, 15-16 min; linear gradient to 100 % A 0 % B to return to the initial conditions before another sample was injected at 20 min. The flow rate was 0.2 mL/min. The Triple TOF 5600 parameters were: nebulizer N_2,_ 50 psi; heating gas, 50 psi; curtain gas, 30 psi; temperature, 500 °C; ion spray voltage floating, -4.5 kV for negative mode and 5.5 kV for positive mode; declustering potential was -60 for negative mode and 60 for positive mode; MS scan range, 50-2000 m/z.

**Transmission electron microscopy observation**

For analysis through transmission electron microscopy, AZ containing proximal parts of pedicel tissues were cut into small portions and fixed in Karnovsky’s fixative solution (2 % paraformaldehyde and 2.5 % glutaraldehyde in 0.1 M sodium phosphate buffer, pH 7.4). Samples were washed with 0.05 M sodium cacodylate buffer and post-fixed for 4 hr in 1 % osmium tetroxide diluted in 0.1 M sodium cacodylate buffer. The samples were then washed with distilled water and stained with 0.5 % uranyl acetate buffer for 16 hr at 4°C. Samples were dehydrated in an increasing ethanol gradient (50 %, 60 %, 70 %, 80 %, 90 %, and 100 %) for 20 min and treated with propylene oxide followed by 1:1, 1:2 propylene oxide: Spurr’s resin solution (10 g of cycloaliphatic epoxide resin (ERL 4221), 6 g of diglycidyl ether of polypropylene glycol (D.E.R. 736), 26 g of nonenyl succinic anhydride, and 0.3 g of dimethylaminoethanol) for 2 hr each. Then, samples were embedded in 100 % Spurr resin solution and sectioned with EM UC7 ultramicrotome (Leica microsystems, Wetzlar, Germany).

**References**

1. Bolger AM, Lohse M, Usadel B. Trimmomatic: a flexible trimmer for Illumina sequence data. Bioinform. 2014:30(15):2114-20

2. Kim D, Langmead B, Salzberg SL. HISAT: a fast spliced aligner with low memory requirements. Nat Methods. 2015:12(4), 357-360.

3. Robinson MD, McCarthy DJ, Smyth GK. edgeR: a Bioconductor package for differential expression analysis of digital gene expression data. Bioinform. 2010:26(1), 139-140.
